# Folate, vitamin B12, and homocysteine status in the Korean population: data from the 2013-2015 Korea National Health and Nutrition Examination Survey

**DOI:** 10.4178/epih.e2024007

**Published:** 2023-12-11

**Authors:** Sihan Song, Bo Mi Song, Hyun-Young Park

**Affiliations:** 1 Division of Population Health Research, Department of Precision Medicine, Korea National Institute of Health, Cheongju, Korea; 2 Korea National Institute of Health, Cheongju, Korea

**Keywords:** Folate, Vitamin B12, Homocysteine, Age, Sex, Korea National Health and Nutrition Examination Survey

## Abstract

**OBJECTIVES:**

We aimed to assess the serum folate, vitamin B12, and homocysteine status in Korean adolescents and adults using national data.

**METHODS:**

Blood samples were collected from participants aged ≥10 years in the Korea National Health and Nutrition Examination Survey 2013-2015. The stored serum samples were used to measure folate, vitamin B12, and homocysteine concentrations. A total of 8,016 participants were included in this analysis. Unweighted descriptive statistics and adjusted geometric means of the B vitamins and homocysteine concentrations were estimated.

**RESULTS:**

Females had higher serum folate and vitamin B12 concentrations and lower serum homocysteine concentrations than males. Folate deficiency (<6.8 nmol/L) and hyperhomocysteinemia (>15 μmol/L) were found in 8.6% and 11.8% of males, respectively. Approximately 3% of males had low or marginally low vitamin B12 status (≤221 pmol/L). Folate and vitamin B12 deficiencies and hyperhomocysteinemia were found in <2% of females. Suboptimal folate status was prevalent among adolescents and young adults, while suboptimal vitamin B12 status and hyperhomocysteinemia were relatively higher in older adults. Adjusted mean homocysteine concentrations were sharply decreased from the first to second decile of serum folate in males.

**CONCLUSIONS:**

In the Korean population, the proportion of males who achieved desirable folate and homocysteine concentrations were lower than those of females. Although most Koreans have adequate vitamin B12, a suboptimal folate status is common, particularly among adolescents and young adults. These findings could establish a foundation for public health initiatives aimed at improving folate levels in the Korean population.

## GRAPHICAL ABSTRACT

[Fig f4-epih-46-e2024007]

## Key Message

Folate and vitamin B12 have significant health impacts throughout the life cycle. However, national-level data on B vitamins in Korea are limited. Serum folate, vitamin B12, and homocysteine concentrations were measured from samples stored during the national survey. In our study, the proportions of folate deficiency and hyperhomocysteinemia were higher in men than in women.Suboptimal folate status was common among adolescents and young adults. Most Koreans had adequate levels of vitamin B12; however, regular monitoring is warranted, especially in the older population. The current data provide a future direction for achieving optimal B vitamin status in the Korean population.

## INTRODUCTION

The water-soluble B vitamins, folate and vitamin B12, are essential for one-carbon metabolism, a network of interrelated biochemical reactions [1]. Folate acts as a cofactor for enzymes involved in DNA and RNA biosynthesis and is involved in supplying methyl groups to the methylation cycle [2]. Vitamin B12 serves as a cofactor in the folate-dependent conversion of homocysteine to methionine and the degradation of methylmalonyl-CoA [3]. Clinical deficiency of either folate or vitamin B12 causes megaloblastic anemia, characterized by large and immature red blood cells [3,4]. Although clinical deficiency is rare, a suboptimal status of folate and/or vitamin B12 may be common and have adverse effects on health [3-5]. Apart from the well-established role of folate and related B vitamins in the prevention of neural tube defects, epidemiological studies have shown protective effects of these vitamins in chronic diseases, including cardiovascular disease (CVD), certain cancers, cognitive dysfunction, and osteoporosis [6,7]. Hyperhomocysteinemia, which results from insufficient folate and vitamin B12, is an independent risk factor for CVD [8].

Inadequate intake is the leading cause of folate and vitamin B12 deficiencies [9]. A wide range of foods, including green leafy vegetables, legumes, some fruits, and cereals, have folate, whereas vitamin B12 is primarily found in animal-source foods [3,4]. Malabsorption of protein-bound vitamin B12 due to gastric atrophy is the predominant cause of vitamin B12 deficiency and depletion in older adults [9]. Increased requirements during pregnancy, neoplastic disease, malabsorptive conditions, and chronic alcoholism are also causative risk factors for folate deficiency [3,9]. A review of national surveys reported that the prevalence of low serum or plasma folate and vitamin B12 vary across the lifespan, exceeding 30% in different subpopulations in some countries [5]. To determine public health impacts, monitoring folate and vitamin B12 status in more representative national surveys is recommended [10]. In Korea, where folic acid fortification is not mandatory, there is limited national-level data on folate, vitamin B12, and their functional indicators [11,12]. Few local studies have evaluated both B vitamins and homocysteine concentrations in Korean adults [13], pregnant and non-pregnant females [14], or older adults [15].

In this study, we aimed to assess serum folate and vitamin B12 status in Korean adolescents and adults using data from the Korea National Health and Nutrition Examination Survey (KNHANES) 2013-2015, for the first time. We also assessed serum homocysteine concentrations, which are functional indicators of folate and vitamin B12 status, and examined their relationship with B vitamins.

## MATERIALS AND METHODS

### Study population

KNHANES, conducted by the Korea Disease Control and Prevention Agency (KDCA), collects nationally representative data on the health and nutritional status of the civilian, non-institutionalized Korean population [16]. In KNHANES 2013-2015, participants underwent a health examination and health interview in a mobile examination center. One week after the initial interview, they were administered a nutrition survey in their homes. During the health examination, blood samples were collected from participants aged ≥ 10 years to obtain laboratory results according to a standard protocol. With consent, these specimens were deposited in the National Biobank of Korea (NBK) for future analyses [16,17]. Frozen specimens from 8,560 participants in KNHANES 2013-2015 were deposited in the NBK. As part of the Biospecimen and Biomarker Program of the NBK, the serum samples stored at -150°C were used to perform biochemical tests, between 2018 and 2020. A total of 8,248 participants with measured serum folate, vitamin B12, and homocysteine concentrations were eligible for this study. Those with impaired kidney function, defined as an estimated glomerular filtration rate (eGFR) below 60 mL/min/1.73 m^2^ (n= 151), and pregnant or lactating females (n= 70) were excluded. Consequently, 376 adolescents aged 10-19 years and 7,640 adults aged ≥ 20 years were included in this study. For the analysis of vitamin B12 status, adults with missing data on serum vitamin B12 (n= 14) were additionally excluded.

### Biochemical methods

Serum folate and vitamin B12 concentrations were measured using an electrochemiluminescence method on a Cobas e602 analyzer (Roche Diagnostics, Basel, Switzerland) and had coefficients of variation (CVs) < 8% and 4%, respectively (internal quality control) [18-20]. Serum homocysteine concentrations were measured using an enzymatic method on a Cobas c702 analyzer (Roche Diagnostics), with CVs < 4%. Serum creatinine concentrations were measured at baseline using the Jaffe rate-blanked and compensated methods using a Hitachi Automatic Analyzer 7600-210 (Hitachi, Tokyo, Japan) [21]. Serum cystatin-C concentrations were measured in stored samples between 2018 and 2020 using an immunoturbidimetric method on a Cobas c702 analyzer (Roche Diagnostics). The stability of the stored samples was evaluated in subsamples by measuring 11-13 biomarkers, which were initially measured at baseline (2013-2015) [18-20]. The values measured at baseline and 5 years later were generally consistent. Moreover, sample loss during storage was not substantial.

In this study, the cut-off for folate deficiency was based on macrocytic anemia as a hematological indicator (deficiency: < 6.8 nmol/L and marginal deficiency: 6.8-13.4 nmol/L) [22]. We also assessed folate status using a cut-off based on homocysteine concentration as a metabolic indicator (< 10 nmol/L) [22,23]. For vitamin B12 status, we used a cut-off of < 148 pmol/L for deficiency and ≤ 221 pmol/L for deficient or marginally deficient status [3]. Cut-offs for homocysteine status were normal (≤ 15 μmol/L) and high (> 15 μmol/L) [24].

### Covariates

Demographic variables for the analysis were categorized as follows: age (10-19, 20-29, 30-39, 40-49, 50-59, 60-69, and ≥ 70 years) and sex. Participants were instructed to fast for at least 8 hours prior to the health examination. The duration of fasting was calculated between the time of venipuncture and the time that the examinee last ate or drank. eGFR was used to evaluate renal function, using the creatinine-cystatin C-based Chronic Kidney Disease Epidemiology Collaboration equation [25]. Renal function was categorized as normal when eGFR was ≥ 90 mL/min/1.73 m^2^ and as mild renal failure when eGFR was between 60 mL/min/1.73 m^2^ and 89 mL/min/1.73 m^2^. In the nutrition survey, participants were asked about their dietary supplement use for at least 2 weeks during the past year.

### Statistical analysis

Sampling weights were not used because our study was a part of KNHANES 2013-2015, which provided consent for specimen storage and future research. Descriptive statistics were used to analyze the characteristics of study participants. The selected percentiles of serum folate, vitamin B12, and homocysteine concentrations were calculated. Geometric means (log-transformed data) were estimated because the distributions of these biomarkers were skewed. Adjusted means and 95% confidence intervals (CIs) of serum folate, vitamin B12, and homocysteine concentrations were estimated by sex across age groups, with adjustments for fasting time and renal function, using the generalized linear model. A p-value for trends was calculated by assigning the median value of the age group to the model as a continuous variable. Analyses were repeated in the subgroups of non-users and users of dietary supplements. The proportions of participants with folate deficiency, vitamin B12 deficiency, and high homocysteine levels were calculated. For folate, the proportions of deficiency and marginal deficiency were calculated according to sex and age groups. For low or marginally low vitamin B12 concentrations and high homocysteine concentrations, data on the age group with the highest proportion was described. To examine the association between B vitamins and homocysteine, we categorized participants into sex-specific deciles of serum folate and vitamin B12 and estimated the geometric means of homocysteine concentrations within each decile adjusting for age, fasting hours, and renal function. Serum folate and vitamin B12 concentrations were mutually adjusted. The p-values for trends were calculated by assigning the median value of each category of B vitamins to the model as a continuous variable. The analyses were repeated for the age subgroups.

All statistical tests and corresponding p-values were two-sided, and p-values < 0.05 were considered statistically significant. All statistical analyses were conducted using SAS version 9.4 (SAS Institute Inc., Cary, NC, USA).

### Ethics statement

This study was approved by the Institutional Review Board (IRB) of the KDCA (IRB No. 2021-04-02-1C-A). KNHANES 2013-2014 was approved by the IRB of the KDCA (2013-07CON-03-4C and 2013-12EXP-03-5C), and KNHANES 2015 was waived by the Bioethics and Safety Act. All the participants provided written informed consent.

## RESULTS

The study population included 49.0% males, 4.7% adolescents aged 10-19 years, and 7.9% older adults aged ≥ 70 years (Table 1). The median age of the participants was 48 years. At the time of venipuncture, 76.4% of participants fasted for ≥ 12 hours. Approximately 80% of participants had normal renal function, and 45.5% of participants in the nutrition survey reported the use of dietary supplements.

Selected percentiles of serum folate, vitamin B12, and homocysteine concentrations are shown in Table 2. The central 90% intervals (5-95th percentiles) were 6.77 nmol/L and 37.62 nmol/L for serum folate, 264 pmol/L and 842 pmol/L for vitamin B12, and 5.98 μmol/L and 15.99 μmol/L for homocysteine. Females had higher serum B vitamins and lower serum homocysteine concentrations than males. These sex differences were observed across age groups (Supplementary Material 1). Adjusted mean serum folate, vitamin B12, and homocysteine concentrations significantly increased with age in both sexes (Table 3). However, young adults aged 20-29 years had relatively higher mean homocysteine concentrations than middle-aged adults. Serum vitamin B12 concentrations were decreased in older individuals. Among those who completed the nutrition survey, the proportion of supplement users was higher in females (50.9%) than in males (39.6%). When stratified by dietary supplement use, associations between age and B vitamins in both sexes were generally consistent with the main analysis (Supplementary Material 2).

**Figure 1.**
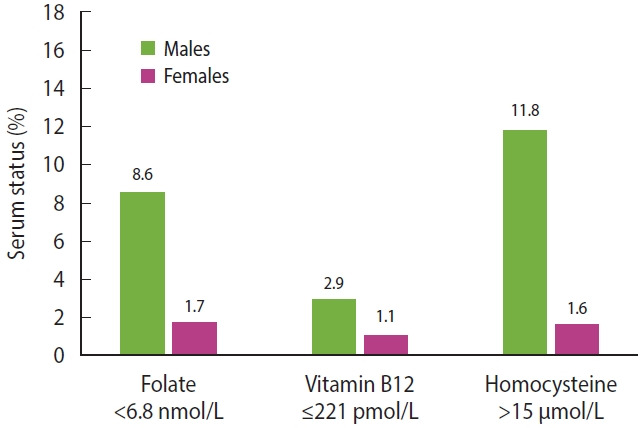
Serum folate, vitamin B12, and homocysteine status in the Korean population.

**Figure 2.**
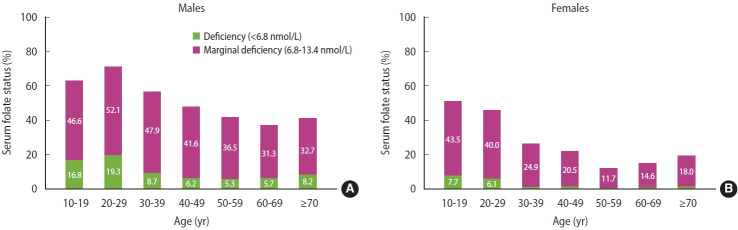
Serum folate status of Korean (A) males and (B) females across age groups.

The proportion of males and females with serum folate concentrations < 6.8 nmol/L were 8.6% and 1.7%, respectively (Figure 1). When the cut-off for folate deficiency based on the metabolic indicator (< 10 nmol/L) was used, 27.1% males and 8.2% females were folate deficient. A suboptimal folate status was common in the Korean population (Figure 2). Among males, over 60% of adolescents and adults aged 20-29 years had deficient or marginal status of serum folate. The prevalence of folate deficiency and marginal deficiency initially declined and then stabilized with increasing age in both sexes. Less than 1% of males and females had vitamin B12 concentrations < 148 pmol/L. Vitamin B12 concentrations ≤ 221 pmol/L were present in 2.9% of males and 1.1% of females. Prevalence of hyperhomocysteinemia (> 15 μmol/L) in males and females were 11.8% and 1.6%, respectively. The highest proportions of vitamin B12 deficiency or marginally deficiency (6.7% in males and 3.4% in females) and hyperhomocysteinemia (20.6% in males and 4.7% in females) were observed among older adults aged ≥ 70 years.

**Figure 3.**
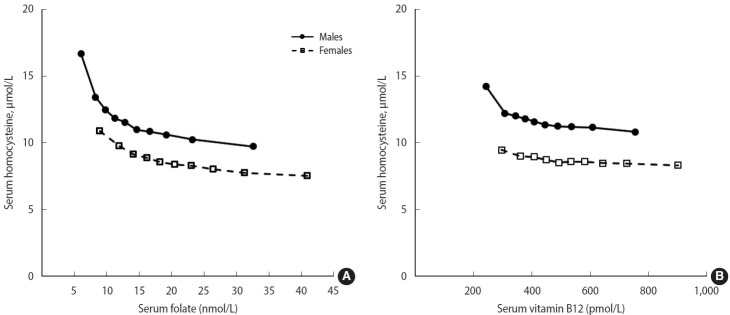
Adjusted means of serum homocysteine according to the deciles of serum (A) folate and (B) vitamin B12 in the Korean population. The geometric means of homocysteine concentrations are plotted against the median values of B vitamins for each decile category adjusted for age, fasting hours, and renal function. Serum folate and vitamin B12 concentrations were mutually adjusted (p for trend <0.001 for each).

In the adjusted models, the highest mean homocysteine concentration was observed in the lowest decile of B vitamins (Figure 3). Among males, a marked decrease in homocysteine concentration was observed from the first to the second decile of folate concentration. A similar but less pronounced pattern was observed in the relationship between vitamin B12 and homocysteine concentrations. Among females, a slight decrease in serum homocysteine concentration was observed with increasing B-vitamin concentrations. Similar inverse relationships were observed in the age subgroup analyses (Supplementary Material 3).

## DISCUSSION

This study presents national-level data regarding serum folate, vitamin B12, and homocysteine concentrations in the Korean population. Serum folate concentrations increased with age and were higher in females than in males. Marginal folate deficiency was common among adolescents and young adults, while most study participants demonstrated adequate levels of vitamin B12. The prevalence of hyperhomocysteinemia was significantly higher in males than in females, with the highest prevalence observed in older males. Our findings indicate that low concentrations of folate and vitamin B12 are significant predictors of elevated homocysteine levels in the Korean population.

Plasma or serum folate and red blood cell folate are useful indicators of folate status at the population level [5,22]. According to the review of national surveys, the prevalence of folate deficiency (< 6.8 nmol/L) was highest in lactating females (49%) in Costa Rica in 1996, prior to folic acid fortification [5,26]. In 1998, the United States and Canada implemented mandatory fortification of cereal-grain products with folic acid [27,28]. As of July 2022, over 90 countries have legislation to mandate cereal grain fortification with folic acid or iron [29]. In many countries, including Costa Rica, the prevalence of folate deficiency markedly decreased after folic acid fortification (prevalence < 5%) [30-32]. Data from the National Health and Nutrition Examination Survey (NHANES), a United States national survey, the prevalence of folate deficiency decreased from 16% pre-fortification (1988-1994) to 0.5% post-fortification (1999-2000) in the population aged ≥ 4 years [31]. Compared with the NHANES study, the median of serum folate concentrations in our study population (16.4 nmol/L) was higher than that in the pre-fortification survey (12.5 nmol/L) but much lower than that in the post-fortification survey (32.2 nmol/L) [31].

Folate status of our study participants was within the range reported in previous studies of Korean adults. In previous studies, approximately 6% to 13% of males [13,33,34] and 0% to 12% of females [13,14,33-36] had serum or plasma folate concentrations < 6.8 nmol/L. Marginal folate deficiency (6.8-13.4 nmol/L) was presented in 44.4% of males and 34.4% of females among 195 Korean adults [13]. Functional folate deficiency was also prevalent, especially in males; in previous studies, 27.9% of males and 4.8% of females among 254 adults aged 19-64 years [34] and 17.2% of males and 5.4% in females among 21,260 adults aged ≥ 40 years had folate concentrations < 10 nmol/L [37]. Folate status was also assessed in a subset of KNHANES 2016-2018 [11]. The distribution of serum folate concentrations across sex and age groups was generally similar to that in our study. In that survey, the median folate concentrations in the Korean population aged ≥ 10 years were 12.5 nmol/L in 3,288 males and 17.0 nmol/L in 3,897 females. Continued monitoring of B vitamins and homocysteine levels in the Korean population is warranted. Moreover, dietary interventions should be considered for individuals who are at high risk of folate deficiency.

In our study, females had significantly higher serum folate concentrations than males and the mean folate concentrations increased with age, with the lowest concentrations observed in adolescents and young adults. Consistent findings were observed in NHANES during both pre-fortification and post-fortification periods. In NHANES 1988-2010, serum folate concentrations were assessed in participants aged ≥ 4 years; mean concentrations were higher in females than in males [38]. In that study, the mean folate concentrations followed U-shaped age patterns, with the lowest concentrations observed in young adults aged 20-39 years [38]. Although circulating folate concentrations are indicators of dietary folate intake, folate metabolism may be altered by biological characteristics. In NHANES 2011-2016, serum folate forms were assessed in the United States population aged ≥ 1 year, which revealed that biologically active folate forms displayed a U-shaped age pattern, while biologically inactive folate increased with age [39]. The higher percentage of females who used supplements may partially explain the sex differences in our study participants. Moreover, our preliminary analysis indicated that the mean daily intake of folate from food (per 1,000 kcal) was higher in females than in males. However, lack of data on folate content of foods and detailed information on dietary supplements limited further analysis. Future research should be conducted on total folate intake from foods and dietary supplements, as well as other lifestyle factors, to better understand the sex differences in folate status.

Plasma or serum vitamin B12 and methylmalonic acid levels serve as the primary indicators of vitamin B12 status. Older individuals are particularly vulnerable to vitamin B12 deficiency due to food-bound cobalamin malabsorption [9,10]. According to the review of nationally representative data, the prevalence of vitamin B12 deficiency was highest in older adults in the United Kingdom (< 150 pmol/L, 31.8%) [5,40]. In NHANES 1994-2004, the prevalence of low serum vitamin B12 among United States adults was 2.9% based on a cut-off of < 148 pmol/L and 10.6% based on < 200 pmol/L; the prevalence of low vitamin B12 status was higher in females than in males and increased with age [41]. In that study, 2% adults aged 19-39 years and 5% adults aged ≥ 65 years, respectively, had vitamin B12 concentrations < 148 pmol/L. Additionally, individuals or people with a low intake of animal-sourced foods are at a high risk of vitamin B12 deficiency [3,9]. For example, in a community-based study of Indian males aged 30-50 years, approximately one-third of the males were lactovegetarians, and 67% of males had plasma vitamin B12 concentrations < 150 pmol/L [42].

In the present study, although suboptimal vitamin B12 status was observed in about 5% of individuals aged ≥ 70 years, severe vitamin B12 deficiency was rare in the overall population. Similarly, data from the Korean Frailty and Aging Cohort Study—a nationwide and multicenter study of community-dwelling older adults aged 70-84 years—revealed that 1.8% and 13.8% of the 2,991 participants had low vitamin B12 concentrations based on the cut-offs of < 148 pmol/L and ≤ 258 pmol/L, respectively [12]. The relatively low prevalence of severe vitamin B12 deficiency in older adults in Korea compared to other countries may be associated with food sources of vitamin B12 and warrants further investigation. For instance, certain non-animal sources of vitamin B12, such as soy-based or vegetable-based fermented foods and seaweeds, are commonly consumed in the Korean population [43,44]. Regular monitoring of both dietary intake and circulating levels of vitamin B12 is warranted, particularly in the older population.

Consistent with previous reports, we observed that serum homocysteine concentrations increased with age, and higher concentrations were observed in males than in females. Homocysteine levels increase with age in both sexes and are usually about 1 µmol/L to 2 µmol/L higher in males than in females [46]. The age-dependent increase in homocysteine may be partially explained by impaired renal function and folate status and food-bound cobalamin malabsorption [1,8,46]. Sex differences in rates of homocysteine remethylation and sex steroids may contribute to the higher homocysteine concentrations in males than in females [46,47]. Beyond biological aspects, homocysteine concentrations are also influenced by a combination of genetic, physiological, and lifestyle factors [46]. In the general population, folate and vitamin B12 deficiencies are the most common causes of elevated homocysteine levels, as the remethylation of homocysteine to methionine is reliant on these vitamins [1]. In the United States, the introduction of folic acid fortification contributed to a significant improvement in homocysteine status; the prevalence estimates for high homocysteine concentrations (> 13 µmol/L) decreased from 13.2% in period before fortification (NHANES 1988-1994) to 4.5% afterwards (NHANES 1999-2000) [48].

In our study, although the prevalence of hyperhomocysteinemia was highest in older adults, relatively higher mean homocysteine concentrations were observed in young adults than in middle-aged adults, which is likely a consequence of the low B vitamin concentrations in young adults. Total homocysteine has been recognized as a functional marker of folate and B vitamin status [13,23]. In the United States, the cut-off values for folate and vitamin B12 deficiencies were established based on serum B vitamin levels below which plasma homocysteine levels were elevated, using data from NHANES 1991-1994; < 10 nmol/L for serum folate and < 300 pmol/L for vitamin B12 [10,23]. Similarly, we observed biphasic patterns of folate and vitamin B12 with homocysteine, with a steep slope at low B vitamin concentrations. These patterns were more pronounced in males than in females, particularly for folate, indicating a higher prevalence of inadequate status among males. The homocysteine data from the present study may guide for future research, as homocysteine serve as an indicator of both B vitamin status and overall health.

To our knowledge, this is the first study to evaluate concentrations of folate, vitamin B12, and homocysteine in Korean adolescents and adults using data from a national survey. However, the biomarkers were measured in a subset of the survey participants and unweighted data were used. Moreover, there is the possibility of residual and unmeasured confounding, including by pathological conditions and lifestyle factors. Nonetheless, the present study offers national-level insights into the distribution and suboptimal status of B vitamins and their functional indicators by sex and age groups in the Korean population. Our data suggest that the proportion of males with desirable folate, vitamin B12, and homocysteine concentrations was lower than that of females. Suboptimal folate levels are particularly prevalent among Korean adolescents and young adults. The relatively higher mean homocysteine concentrations in young adults than in middle-aged adults warrant further assessment of B vitamin status. This analysis should include data on red blood cell folate and detailed information on B vitamin intake from food and supplements. Our results underscore the importance of regular nutritional monitoring of B vitamin status in a more representative setting and may serve as a basis for public health efforts to improve folate levels in the Korean population.

## Figures and Tables

**Figure f4-epih-46-e2024007:**
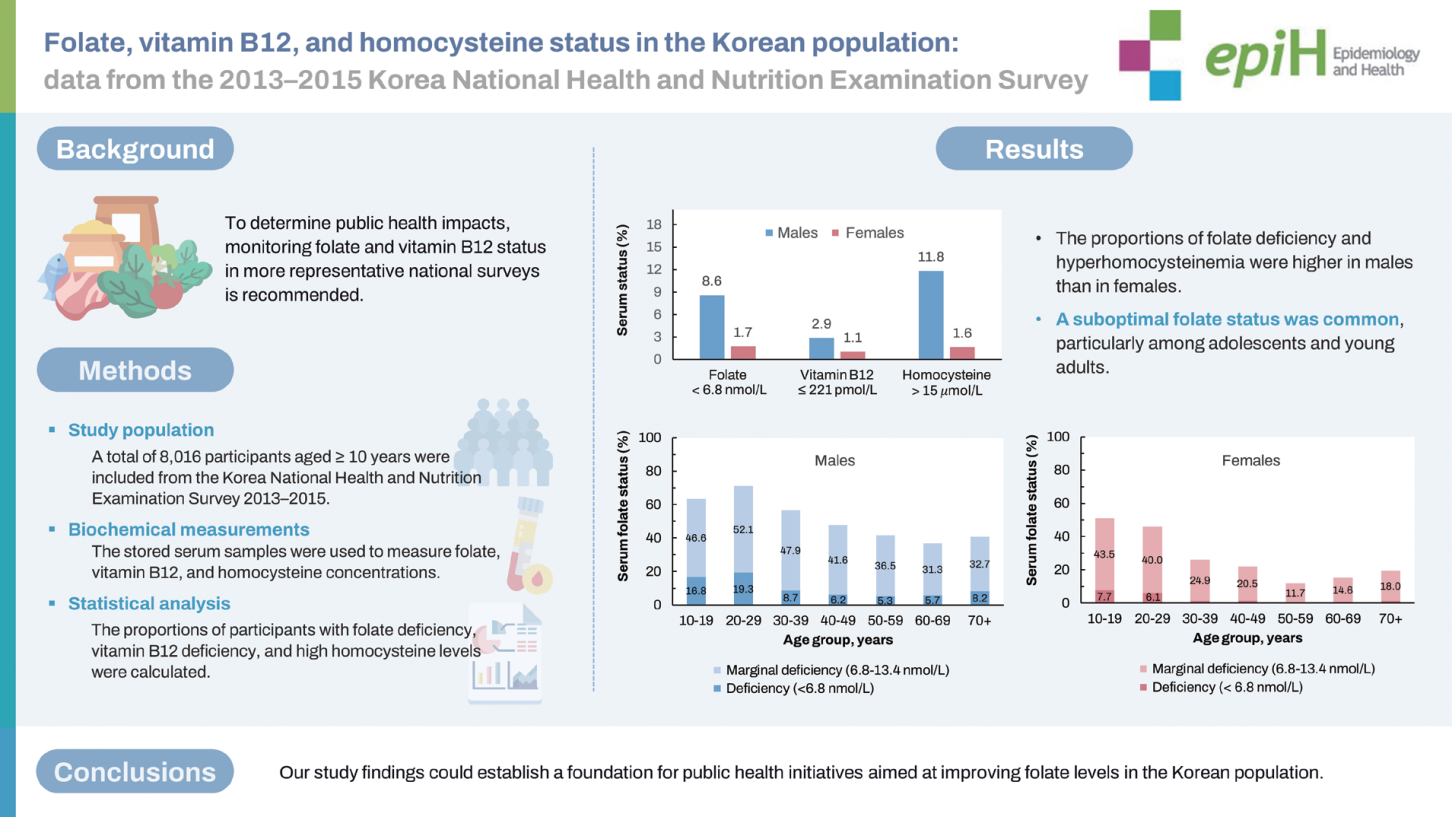


**Table 1.** Characteristics of study participants

Characteristics	n (%)
Sex	
Male	3,931 (49.0)
Female	4,085 (51.0)
Age (yr)	
10-19	376 (4.7)
20-29	870 (10.8)
30-39	1,394 (17.4)
40-49	1,601 (20.0)
50-59	1,781 (22.2)
60-69	1,364 (17.0)
≥70	630 (7.9)
Fasting time (hr)	
<8	156 (1.9)
8-12	1,739 (21.7)
≥12	6,121 (76.4)
Renal function	
Normal (eGFR ≥90)	6,408 (79.9)
Mild loss (eGFR 60-90)	1,608 (20.1)
Dietary supplement use^1^	
No	3,882 (54.5)
Yes	3,245 (45.5)

eGFR, estimated glomerular filtration rate.

1 Among participants who completed the nutrition survey (n=7,127).

**Table 2.** Distribution of serum folate, vitamin B12, and homocysteine concentrations by sex in the Korean population

Serum concentrations	n	GM (95% CI)	Selected percentiles						
			5th	10th	25th	50th	75th	90th	95th
Folate (nmol/L)									
All	8,016	16.28 (16.09, 16.47)	6.77	8.36	11.45	16.40	23.14	31.82	37.62
Male	3,931	13.68 (13.46, 13.90)	5.89	7.11	9.69	13.68	19.16	26.61	32.62
Female	4,085	19.25 (18.97, 19.53)	8.79	10.55	14.02	19.23	26.41	35.11	41.06
Vitamin B12 (pmol/L)									
All	8,002	473 (469, 477)	264	306	374	472	595	735	842
Male	3,925	431 (426, 436)	244	284	345	427	537	665	756
Female	4,077	518 (512, 524)	298	338	409	513	646	793	904
Homocysteine (µmol/L)									
All	8,016	9.51 (9.44, 9.57)	5.98	6.56	7.69	9.29	11.26	13.75	15.99
Male	3,931	11.16 (11.05, 11.27)	7.48	8.02	9.22	10.69	12.74	15.60	19.24
Female	4,085	8.14 (8.08, 8.21)	5.62	6.03	6.92	7.98	9.43	11.12	12.46

GM, geometric mean; CI, confidence interval.

**Table 3.** Adjusted^1^ means of serum folate, vitamin B12, and homocysteine by sex and age in the Korean population

Variables	n	Folate (nmol/L)	n	Vitamin B12 (pmol/L)	n	Homocysteine (µmol/L)
Male						
Age (yr)						
10-19	208	10.35 (9.59, 11.17)	208	395 (373, 418)	208	11.82 (11.27, 12.39)
20-29	457	9.51 (8.97, 10.08)	456	376 (360, 392)	457	13.24 (12.77, 13.73)
30-39	680	11.56 (10.98, 12.16)	680	408 (393, 424)	680	12.00 (11.63, 12.39)
40-49	760	12.95 (12.33, 13.60)	759	423 (408, 439)	760	11.84 (11.49, 12.21)
50-59	792	14.05 (13.40, 14.73)	791	438 (423, 453)	792	11.78 (11.44, 12.13)
60-69	704	15.20 (14.49, 15.95)	702	459 (443, 476)	704	11.75 (11.41, 12.11)
≥70	330	15.62 (14.67, 16.63)	329	418 (399, 438)	330	12.44 (11.97, 12.93)
p for trend		<0.001		<0.001		0.003
Female						
Age (yr)						
10-19	168	12.78 (11.78, 13.87)	168	467 (436, 500)	168	8.81 (8.45, 9.19)
20-29	413	13.66 (12.85, 14.52)	413	452 (430, 476)	413	9.27 (8.98, 9.57)
30-39	714	17.20 (16.30, 18.16)	713	512 (490, 536)	714	8.31 (8.08, 8.54)
40-49	841	18.33 (17.40, 19.31)	841	511 (490, 534)	841	8.41 (8.18, 8.64)
50-59	989	21.06 (20.04, 22.14)	986	552 (529, 575)	989	8.66 (8.44, 8.88)
60-69	660	20.83 (19.79, 21.93)	658	541 (519, 565)	660	9.21 (8.97, 9.45)
≥70	300	20.35 (19.09, 21.69)	298	509 (483, 537)	300	9.57 (9.26, 9.89)
p for trend		<0.001		<0.001		0.001

Values are presented as geometric mean (95% confidence interval).

1 Adjusted for fasting hours and renal function.

## Supplementary materials

Supplementary Material 1. Distribution of serum folate, vitamin B12, and homocysteine concentrations by sex and age in the Korean population

Supplementary Material 2. Adjusted means of serum folate, vitamin B12, and homocysteine concentrations by sex and age, stratified by dietary supplement use

Supplementary Material 3. Adjusted means of serum homocysteine according to deciles of serum folate and vitamin B12 by sex and age in the Korean population.
