# Supplementary material for: Folate, vitamin B12, and homocysteine status in the Korean population: data from the 2013-2015 Korea National Health and Nutrition Examination Survey
Source: Epidemiol Health. 2023 Dec 11;46:e2024007. doi: 10.4178/epih.e2024007 (PMC10928471; doi:10.4178/epih.e2024007)
Supplement: Supplementary Material 1. — Distribution of serum folate, vitamin B12, and homocysteine concentrations by sex and age in the Korean population [file epih-46-e2024007-Supplementary-1.docx]

**Supplementary Material 1.** Distribution of serum folate, vitamin B12, and homocysteine concentrations by sex and age in the Korean population

| Participants |  | N | Selected percentiles | | | | | | | |
| --- | --- | --- | --- | --- | --- | --- | --- | --- | --- | --- |
|  |  |  | 5th | 10th | 25th | 50th | 75th | 90th | 95th |  |
| *Folate, nmol/L* | | | | | | | | | | |
| Male | Age group, years |  |  |  |  |  |  |  |  |  |
|  | 10–19 | 208 | 4.85 | 5.71 | 7.92 | 11.33 | 15.69 | 23.08 | 27.25 |  |
|  | 20–29 | 457 | 4.73 | 5.64 | 7.41 | 10.24 | 14.25 | 19.57 | 22.67 |  |
|  | 30–39 | 680 | 5.96 | 7.09 | 9.14 | 12.41 | 17.09 | 22.43 | 27.50 |  |
|  | 40–49 | 760 | 6.34 | 7.68 | 10.37 | 13.79 | 18.39 | 25.31 | 31.76 |  |
|  | 50–59 | 792 | 6.66 | 8.24 | 10.65 | 14.85 | 20.53 | 27.32 | 33.86 |  |
|  | 60–69 | 704 | 6.52 | 7.97 | 10.92 | 16.38 | 22.30 | 31.10 | 36.13 |  |
|  | 70+ | 330 | 5.82 | 7.68 | 10.67 | 15.10 | 23.58 | 33.71 | 39.41 |  |
| Female | Age group, years |  |  |  |  |  |  |  |  |  |
|  | 10–19 | 168 | 6.18 | 7.27 | 9.45 | 13.30 | 18.98 | 24.87 | 26.79 |  |
|  | 20–29 | 413 | 6.36 | 8.24 | 10.92 | 14.16 | 19.37 | 26.27 | 31.21 |  |
|  | 30–39 | 714 | 8.79 | 10.22 | 13.27 | 17.87 | 24.60 | 32.98 | 38.30 |  |
|  | 40–49 | 841 | 9.42 | 10.89 | 14.04 | 19.39 | 26.21 | 33.14 | 37.33 |  |
|  | 50–59 | 989 | 10.83 | 12.66 | 16.63 | 21.74 | 29.38 | 37.98 | 43.19 |  |
|  | 60–69 | 660 | 10.16 | 11.96 | 16.23 | 21.35 | 28.77 | 38.39 | 43.58 |  |
|  | 70+ | 300 | 9.23 | 11.51 | 14.77 | 19.63 | 29.58 | 41.44 | 56.68 |  |
| *Vitamin B12, pmol/L* | | | | | | | | | | |
| Male | Age group, years |  |  |  |  |  |  |  |  |  |
|  | 10–19 | 208 | 242 | 276 | 328 | 416 | 499 | 629 | 706 |  |
|  | 20–29 | 456 | 224 | 264 | 318 | 381 | 480 | 568 | 627 |  |
|  | 30–39 | 680 | 252 | 284 | 339 | 417 | 513 | 628 | 720 |  |
|  | 40–49 | 759 | 254 | 296 | 351 | 426 | 531 | 632 | 702 |  |
|  | 50–59 | 791 | 254 | 292 | 359 | 449 | 562 | 695 | 776 |  |
|  | 60–69 | 702 | 255 | 301 | 362 | 460 | 596 | 731 | 853 |  |
|  | 70+ | 329 | 202 | 245 | 329 | 436 | 570 | 680 | 852 |  |
| Female | Age group, years |  |  |  |  |  |  |  |  |  |
|  | 10–19 | 168 | 277 | 312 | 378 | 474 | 577 | 716 | 802 |  |
|  | 20–29 | 413 | 275 | 309 | 372 | 441 | 559 | 680 | 735 |  |
|  | 30–39 | 713 | 309 | 347 | 413 | 514 | 635 | 761 | 854 |  |
|  | 40–49 | 841 | 306 | 341 | 414 | 507 | 626 | 749 | 852 |  |
|  | 50–59 | 986 | 318 | 354 | 440 | 548 | 683 | 841 | 953 |  |
|  | 60–69 | 658 | 286 | 339 | 418 | 547 | 684 | 883 | 969 |  |
|  | 70+ | 298 | 244 | 322 | 382 | 494 | 618 | 832 | 1021 |  |
| *Homocysteine, 𝜇mol/L* | | | | | | | | | | |
| Male | Age group, years |  |  |  |  |  |  |  |  |  |
|  | 10–19 | 208 | 6.29 | 6.90 | 8.23 | 9.84 | 11.59 | 16.08 | 26.92 |  |
|  | 20–29 | 457 | 7.56 | 8.11 | 9.45 | 11.11 | 13.31 | 17.09 | 25.26 |  |
|  | 30–39 | 680 | 7.39 | 7.81 | 8.97 | 10.30 | 11.72 | 14.68 | 18.12 |  |
|  | 40–49 | 760 | 7.53 | 8.01 | 9.12 | 10.37 | 12.24 | 14.33 | 17.39 |  |
|  | 50–59 | 792 | 7.56 | 8.04 | 9.17 | 10.69 | 12.60 | 15.02 | 17.56 |  |
|  | 60–69 | 704 | 7.59 | 8.19 | 9.40 | 10.95 | 13.12 | 16.10 | 18.90 |  |
|  | 70+ | 330 | 8.31 | 8.80 | 10.28 | 11.91 | 14.47 | 17.86 | 22.67 |  |
| Female | Age group, years |  |  |  |  |  |  |  |  |  |
|  | 10–19 | 168 | 5.31 | 5.69 | 6.64 | 7.97 | 9.35 | 10.83 | 12.58 |  |
|  | 20–29 | 413 | 5.61 | 6.17 | 7.09 | 8.26 | 9.56 | 11.60 | 13.63 |  |
|  | 30–39 | 714 | 5.46 | 5.80 | 6.44 | 7.44 | 8.54 | 9.90 | 10.97 |  |
|  | 40–49 | 841 | 5.39 | 5.81 | 6.56 | 7.50 | 8.72 | 10.24 | 11.19 |  |
|  | 50–59 | 989 | 5.71 | 6.05 | 6.92 | 7.90 | 9.29 | 10.75 | 11.83 |  |
|  | 60–69 | 660 | 6.23 | 6.73 | 7.64 | 8.81 | 10.21 | 12.03 | 13.51 |  |
|  | 70+ | 300 | 6.97 | 7.27 | 8.22 | 9.46 | 11.18 | 13.23 | 14.86 |  |
