# Supplementary material for: Folate, vitamin B12, and homocysteine status in the Korean population: data from the 2013-2015 Korea National Health and Nutrition Examination Survey
Source: Epidemiol Health. 2023 Dec 11;46:e2024007. doi: 10.4178/epih.e2024007 (PMC10928471; doi:10.4178/epih.e2024007)
Supplement: Supplementary Material 2. — Adjusted means of serum folate, vitamin B12, and homocysteine concentrations by sex and age, stratified by dietary supplement use [file epih-46-e2024007-Supplementary-2.docx]

**Supplementary Material 2.** Adjusted means of serum folate, vitamin B12, and homocysteine concentrations by sex and age, stratified by dietary supplement use

|  | Male | | | |  | Female | | | |
| --- | --- | --- | --- | --- | --- | --- | --- | --- | --- |
| Geometric means (95% CIs) |  | Nonusers (n = 2,032) |  | Users (n = 1,330) |  |  | Nonusers (n = 1,850) |  | Users (n = 1,915) |
| Folate, nmol/L |  |  |  |  |  |  |  |  |  |
| Age, years |  |  |  |  |  |  |  |  |  |
| 10–19 | 131 | 9.47 (8.63, 10.38) | 50 | 14.92 (12.71, 17.51) |  | 109 | 11.52 (10.47, 12.68) | 34 | 17.50 (14.71, 20.81) |
| 20–29 | 280 | 8.96 (8.34, 9.63) | 104 | 12.24 (10.82, 13.84) |  | 235 | 12.32 (11.41, 13.30) | 125 | 16.04 (14.41, 17.86) |
| 30–39 | 329 | 11.01 (10.28, 11.79) | 229 | 13.50 (12.22, 14.93) |  | 363 | 14.55 (13.56, 15.61) | 299 | 21.39 (19.63, 23.30) |
| 40–49 | 365 | 12.00 (11.25, 12.80) | 244 | 15.52 (14.09, 17.09) |  | 376 | 15.37 (14.34, 16.47) | 413 | 22.30 (20.57, 24.18) |
| 50–59 | 399 | 12.78 (12.02, 13.59) | 279 | 17.22 (15.70, 18.89) |  | 341 | 18.15 (16.95, 19.43) | 568 | 24.10 (22.33, 26.01) |
| 60–69 | 356 | 13.43 (12.61, 14.29) | 285 | 18.60 (17,00, 20.35) |  | 291 | 18.43 (17.21, 19.74) | 328 | 24.33 (22.48, 26.32) |
| 70+ | 172 | 13.12 (12.10, 14.24) | 139 | 19.57 (17.53, 21.85) |  | 135 | 17.77 (16.34, 19.32) | 148 | 24.17 (21.92, 26.65) |
| P for trend |  | <0.001 |  | <0.001 |  |  | <0.001 |  | <0.001 |
| Vitamin B12, pmol/L |  |  |  |  |  |  |  |  |  |
| Age, years |  |  |  |  |  |  |  |  |  |
| 10–19 | 131 | 383 (356, 411) | 50 | 470 (417, 529) |  | 109 | 456 (418, 498) | 34 | 542 (472, 622) |
| 20–29 | 280 | 358 (339, 378) | 104 | 451 (412, 494) |  | 235 | 452 (421, 484) | 125 | 459 (421, 499) |
| 30–39 | 329 | 398 (377, 419) | 229 | 440 (408, 474) |  | 363 | 504 (473, 537) | 298 | 527 (493, 565) |
| 40–49 | 365 | 417 (397, 439) | 243 | 465 (433, 499) |  | 376 | 487 (457, 519) | 413 | 531 (498, 566) |
| 50–59 | 398 | 427 (408, 448) | 279 | 482 (450, 516) |  | 339 | 527 (495, 561) | 567 | 574 (541, 610) |
| 60–69 | 356 | 449 (428, 472) | 284 | 492 (460, 526) |  | 290 | 519 (487, 552) | 327 | 558 (524, 595) |
| 70+ | 172 | 395 (371, 421) | 138 | 468 (431, 508) |  | 134 | 474 (439, 511) | 147 | 547 (506, 591) |
| P for trend |  | <0.001 |  | 0.018 |  |  | <0.001 |  | <0.001 |
| Homocysteine, 𝜇mol/L |  |  |  |  |  |  |  |  |  |
| Age, years |  |  |  |  |  |  |  |  |  |
| 10–19 | 131 | 12.50 (11.73, 13.33) | 50 | 9.60 (8.79, 10.48) |  | 109 | 9.08 (8.59, 9.61) | 34 | 7.45 (6.87, 8.09) |
| 20–29 | 280 | 13.66 (12.99, 14.36) | 104 | 11.59 (10.84, 12.40) |  | 235 | 9.61 (9.18, 10.06) | 125 | 8.70 (8.27, 9.15) |
| 30–39 | 329 | 12.29 (11.72, 12.89) | 229 | 11.33 (10.73, 11.97) |  | 363 | 8.59 (8.24, 8.96) | 299 | 7.91 (7.60, 8.24) |
| 40–49 | 365 | 12.30 (11.76, 12.86) | 244 | 10.93 (10.37, 11.53) |  | 376 | 8.88 (8.52, 9.25) | 413 | 7.93 (7.63, 8.23) |
| 50–59 | 399 | 12.14 (11.63, 12.66) | 279 | 10.85 (10.32, 11.41) |  | 341 | 9.02 (8.66, 9.39) | 568 | 8.31 (8.02, 8.61) |
| 60–69 | 356 | 12.13 (11.62, 12.67) | 285 | 11.02 (10.49, 11.57) |  | 291 | 9.39 (9.01, 9.78) | 328 | 8.98 (8.65, 9.32) |
| 70+ | 172 | 13.61 (12.86, 14.40) | 139 | 11.04 (10.40, 11.72) |  | 135 | 9.98 (9.50, 10.49) | 148 | 9.15 (8.73, 9.57) |
| P for trend |  | 0.074 |  | 0.905 |  |  | 0.107 |  | <0.001 |

Abbreviation: CIs, confidence intervals.

Adjusted for fasting hours and renal function.
