# Supplementary material for: Folate, vitamin B12, and homocysteine status in the Korean population: data from the 2013-2015 Korea National Health and Nutrition Examination Survey
Source: Epidemiol Health. 2023 Dec 11;46:e2024007. doi: 10.4178/epih.e2024007 (PMC10928471; doi:10.4178/epih.e2024007)
Supplement: Supplementary Material 3. — Adjusted means of serum homocysteine according to deciles of serum folate and vitamin B12 by sex and age in the Korean population. [file epih-46-e2024007-Supplementary-3.docx]

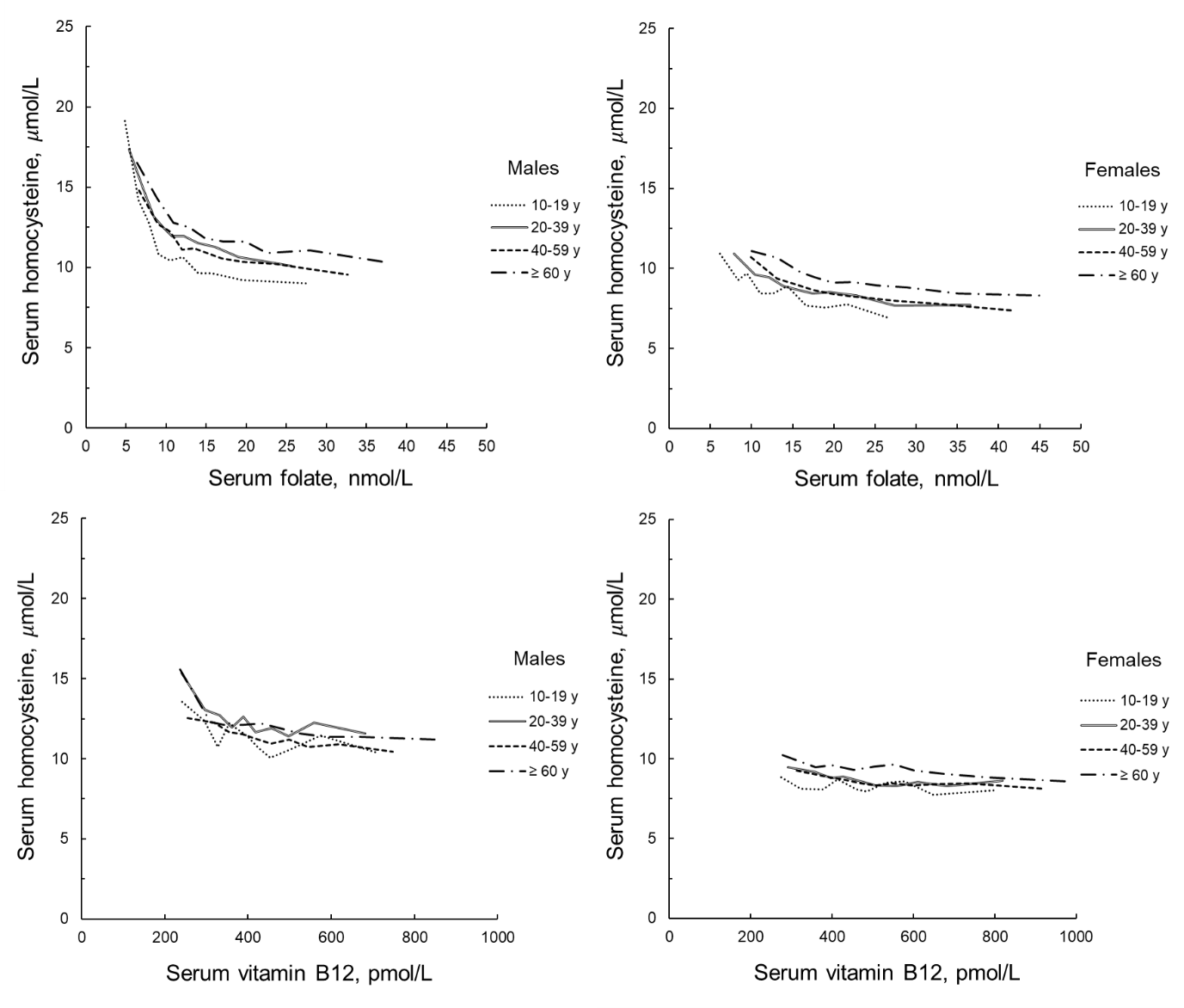


**Supplementary Material 3.** Adjusted means of serum homocysteine according to deciles of serum folate and vitamin B12 by sex and age in the Korean population.

The geometric means of homocysteine concentrations are plotted against the median values of B vitamins for each decile category. Adjusted for age (within age group), fasting hours, and renal function. Serum folate and vitamin B12 concentrations were mutually adjusted. The associations were statistically significant, except for the relationship between vitamin B12 and homocysteine levels in women aged 10–19 years.
